# Evasion of “mandatory” social health insurance for the formal sector: evidence from Lao PDR

**DOI:** 10.1186/s12913-015-1132-5

**Published:** 2015-10-19

**Authors:** Sarah Alkenbrack, Kara Hanson, Magnus Lindelow

**Affiliations:** World Bank Group, Washington, DC USA; London School of Hygiene and Tropical Medicine, London, UK

**Keywords:** Health insurance, Social security, Social health insurance, Private sector, Lao PDR, South-east Asia

## Abstract

**Background:**

In the last decade, almost every low- or middle-income country in the world has expressed support for universal health coverage (UHC). While at the beginning of the UHC movement, country strategies focused on increasing access to the formal sector as the first step of UHC, there is now consensus that countries should cover the entire population, with particular attention to covering the poor. However, it is often assumed that mandatory schemes will automatically cover their target populations, and consequently little is known about why firms comply or do not comply with enrolment requirements.

Using the experience of Lao PDR, where the enrolment rate in the mandatory social security scheme is low and the capacity for regulation is weak, we conducted this study to better understand the determinants of enrolment of private sector firms in mandatory social security.

**Methods:**

We used a cross-sectional case-comparison design, surveying 130 firms. We applied a structured questionnaire to explore determinants of enrolment, specifically looking at firm characteristics (e.g., industry category, ownership); sociodemographic characteristics of company heads; firms’ risk perceptions; details of employment contracts; employee benefits; and exposure to social security. Closed ended questions were analysed quantitatively, while content analysis was applied to open-ended questions. Logistic regression was used to examine the determinants of enrolment.

**Results:**

Smaller privately owned firms in the services industry were the least likely to enrol in social security, while firms in the trade industry were more likely to enrol than firms in manufacturing, construction, or services. The main reason for not enrolling was that firms offered a better package of benefits to their employees, although further investigation of company benefits showed that this was not the case in practice. Additional reasons for non-compliance were lack of knowledge and poor quality of care at government hospitals.

**Conclusions:**

The study contributes to the dialogue on how best to increase coverage in the formal sector, which is an important element of achieving UHC. It also provides much needed information about the motivation of private sector firms to comply with mandatory schemes.

## Background

In the last decade, low- and middle-income countries have made considerable progress towards the goal of universal health coverage (UHC). While each country’s pathway toward UHC depends on the historical structure of the health care financing system, there is an expectation that in countries with mandatory social health insurance (SHI) for the private sector, increasing coverage of SHI will be fairly straightforward given the relative ease with which contributions can be collected and employees can be identified. However, in reality, many countries face challenges enrolling formal private sector firms, even where enrolment is mandatory.

This study explores the case of Lao PDR where a social security scheme for formal private sector firms was introduced in 1999. As in many countries, the social security scheme in Lao PDR includes health and non-health benefits, and covers employees of private companies (including previously owned state-owned enterprises) and their dependents. Because the social security scheme is mandatory and includes a SHI component, the Government of Lao PDR views enrolment in social security as an important pathway towards achieving universal health coverage. However, despite its mandatory nature, the social security scheme has suffered from low coverage. This low coverage is due to two major factors: a small proportion of private sector firms have been targeted for enrolment; and within the target group, few firms comply with the mandatory enrolment law. Among the firms targeted, there is little understanding as to which firms enrol, and which firm characteristics are associated with enrolment. If the Government of Lao PDR is to make progress in expanding health coverage through social security, a greater understanding is needed about the factors influencing enrolment in the scheme.

This study *explores the firm-level determinants of enrolment in social security.* The study also examines several other aspects of enrolment, including the motivation of employers to enrol/not enrol in social security, firms’ experiences with the scheme, and the extent to which firms are employing strategies to evade social security contributions. The findings from the analysis shed light on the prospects for expanding coverage of social security in Lao PDR and other countries facing challenges of getting the private sector to enrol in mandatory SHI or social security schemes.

## Review of the literature

There has been little study of the factors affecting the decision to enrol in social security or social health insurance at the firm level, probably because the mandatory nature of such schemes theoretically eliminates the decision-making process. However, in reality, most developing countries face difficulty getting the formal sector to enrol in SHI: compliance is low, enforcement is weak, and several evasion tactics are commonly used [1, 2]. It is therefore useful to know why some firms comply with mandatory insurance policies, while others do not. Three bodies of literature shed light on the factors affecting firms’ enrolment in insurance.

Studies examining compliance with social security offer some insight into firms’ compliance with social security laws, but typically focus on *how* and *why* firms evade contributions [1–6]. One study from Shanghai examined the factors affecting compliance with social security. The study used a dataset of 2,200 firms to explore the determinants of enrolment and found that larger firms and firms in the real estate or construction industries were less likely to comply with social security obligations, but that a firm’s risk profile, i.e. how dangerous the business is, was not associated with compliance.

Literature on private employer-based health care in the United States (US) offers insight into firms’ preferences for employer-based health insurance, although the mechanism for insuring these firms is voluntary. This body of literature is relevant because enrolment in social security is effectively “voluntary” in countries where enforcement is weak. A 2004 review [7] summarized much of the literature in this area and showed that the following types of firm were more likely to provide health insurance: unionized firms; firms in the manufacturing industry [8]; larger firms; firms with higher wage workers [9]; and firms with lower staff turnover rates and age heterogeneity [10].

Finally, a third body of literature looks at reasons for participating in formal schemes, defining formality as the decision to participate in societal institutions, e.g., national and local treasuries; governmental programs; banking systems; trade organizations; social security; etc. Levenson and Maloney (1988) offer a framework for explaining when and how institutions will participate in societal institutions [11]. Using data from urban firms in Mexico they showed that larger (measured by revenues) and older firms were more likely to register with the federal treasury, the social security administration (IMSS), and to be enumerated in the Census of firms. A study from Peru supports these findings, although does not specifically examine participation in social security [12].

In summary, these bodies of evidence shed light on factors that are likely to shape firms’ decision to enroll in social security, but there is still little known about the determinants of enrolment in social security at the firm level.

### The setting

Like most other low- and middle-income country governments, the Government of Laos is moving towards universal health coverage. The health financing strategy outlines a plan to expand coverage through four schemes: the civil servants’ State Authority for Social Security (SASS); a mandatory Social Health Insurance scheme for private and state-owned enterprises, run by the Social Security Organization (SSO); voluntary community-based health insurance (CBHI) for the informal sector and self-employed workers; and health equity funds (HEFs) for households living in extreme poverty. Additionally, the government subsidizes care and has been piloting a free maternal health care initiative for women and children 5 years of age and younger. However, health insurance coverage in the country is still low: in 2011, only 18.5 % of the population was enrolled in health insurance [13]. For the remainder of the population, they either pay out-of-pocket for health care, or forego care altogether. The challenges of expanding health insurance coverage in Lao PDR have been discussed elsewhere, but include a small formal sector, challenges getting informal sector households to enroll in CBHI; lack of trust in the health care system; and other factors [14, 15].

The Social Health Insurance scheme is part of a larger social security program targeting employees in the private formal sector (including previously state-owned enterprises) and their dependents. The scheme is managed by the SSO - a semi-autonomous organization within the Ministry of Labor and Social Welfare, and is financed through a combination of employee and employer contributions.^1^ The full package of social security benefits includes medical care, paid sick leave, paid maternity leave, death benefits, employment injury or occupational disease benefits, retirement pensions, life insurance, and disability insurance. Upon enrolment in social security, a firm is automatically enrolled in the various social security benefits and therefore, enrolment in social health insurance is linked to enrolment in the other benefit packages by design. However, the health care fund, which also receives government subsidies, is the largest benefit and finances outpatient and inpatient care, and prescription drugs available at hospitals. There are no co-payments or limits on the number of contacts or services provided. Public providers are paid by capitation, which, at the time of the study, was fixed at LAK 80,000 (US$ 9.40) per insured person per year [16].

Although enrolment in social security was mandatory for all enterprises with at least 10 employees at the time of the study, coverage has remained low since the scheme’s inception.^2^ Low enrolment can be attributed, at least in part, to the small scale of the formal sector (roughly 5 % of the population), limited geographic reach of the scheme (4 provinces only), lack of enforcement and weak regulatory structure, and limited capacity of the SSO to attract new members through scheme promotion. Despite the mandatory law, there has been little enforcement of the scheme [17]. The lack of enforcement is due to a variety of factors. First, until recently, enrolment was mandated through a Ministerial decree (SSO Decree 207), which is weaker than a law promulgated by the President. Second, there are no regulatory procedures to enact penalties for non-compliant firms. Although an inspection unit at SSO was introduced in 2008 with a mandate of identifying non-compliant enterprises, at the time of the study, only verbal warnings could be issued. Furthermore, the SSO office has traditionally been understaffed, with very little capacity to carry out inspections. As a step to strengthening the regulatory framework around social security, the SSO promulgated a law specific to social security in 2013 and it was implemented in 2014. However, it is not clear to what extent the new law is enforced or whether regulatory procedures, including penalties for non-compliance, accompany the new law. In summary, the lack of enforcement of social security at the time of the study made compliance with the scheme somewhat “voluntary” for firms, justifying the need to understand the factors affecting enrolment.

## Methods

We used a cross-sectional case-comparison design of 130 firms, of which 65 were enrolled and 65 non-enrolled. The study was conducted in Vientiane Capital, the province with the highest concentration of formal sector enterprises. A structured questionnaire with both closed- and open-ended questions was administered to employers. Other methods were employed to help set the context for the study and to shed light on the potential for expanding coverage. These methods include: reviews of legislation and documents; key informant interviews with SSO staff members and private employers; secondary data analysis of the Lao Economic Census; and Excel projections of coverage expansion. The purpose of the interviews with SSO staff members was to learn more about the scheme, its implementation, and the SSO; to identify the optimal sampling frame for the study; and to better understand how the target group for the scheme was defined. Private employer interviews helped to understand the factors influencing enrolment in social security. This pre-survey work was exploratory in nature and no formal methods were used to analyse findings.

### Sample selection

We used the list of firms maintained by the SSO, herein referred to as the *SSO database*, as the sampling frame for this study. We restricted the sample to firms in Vientiane Capital, as they represent the large majority of the firms in the sampling frame. This restricted sampling frame included 1320 firms (388 member and 932 non-member firms). We randomly selected 130 employers (including 65 member firms and 65 non-member firms) from the four biggest industries in the SSO target group: manufacturing; construction; trade; and services, using stratified random sampling, stratifying by industry and firm size (10–19 employees; 20–49 employees; 50–99 employees; and 100 or more employees), making a total of 16 strata in the SSO-member group and 16 strata in the non-member group. Selecting a subsample of industries is consistent with the methodology used in the World Bank Enterprise Surveys [18]. Once the firms were selected, they were contacted by telephone to request participation in the survey. Following acceptance, an appointment was made for a face-to-face interview. If the prospective interviewee was not available, the interviewers called back up to three times to reschedule the appointment. If firms refused, a replacement firm was selected from the same stratum, to ensure that 65 firms in each group (SSO and non-SSO) were interviewed.

It is important to note that the SSO sample represents only a fraction of firms that should be eligible for social security in Laos because the SSO obtains its list of target firms from the central Tax Registration office, which does not include all firms in the country. For example, some firms that are “formal” (i.e., they pay taxes and have a permanent location), pay taxes to other entities and are therefore not targeted by the SSO. Additionally, the Labor Law mandates enrolment of social security but for “all firms with at least one or more employees”. These smaller or less formalized firms were not targeted by the SSO at the time of the study [19].

### Data collection

A structured questionnaire was administered to heads of companies, i.e., chief executive officer, director, general manager, etc. where possible. Closed-ended questions were designed to capture variables that are expected to influence enrolment in social security, including: characteristics of businesses (industry category, ownership, company size, revenues, percentage of permanent employees, percentage of female workforce, age distribution, number of service outlets/branches, membership of a business organization, and level where tax payment is made); sociodemographic characteristics of heads of company (education, age, nationality, gender); risk perceptions of firms; details of employment contracts; employee benefits; and awareness of, and experience with, social security. For some questions, employers were asked to obtain information from company records if available, e.g., the number of employees by employment contract; and the amount paid to social security and other benefits. Open-ended questions were also included in the questionnaire to capture qualitative information that could be used to help interpret findings.

A local research team was hired to carry out data collection. The questionnaire and study protocol were approved by the SSO and ethical approval was granted by the National Institute of Public Health in Laos and the London School of Hygiene and Tropical Medicine in the United Kingdom. Pilot-testing took place in February 2009 and field work took place from March through May 2009.

### Analysis

Analysis of quantitative information from the closed-ended questions was performed in Stata 10.0. Much of the data analysis was descriptive but a logit model was used to understand the determinants of enrolment. The logit model is represented by the following equation, where P_SS_ represents the probability of enrolling in social security:

$$ {\mathrm{P}}_{\mathrm{SS}}=\kern0.75em 1/{\left(1+\mathrm{e}\right)}^{-\left({b}_0+{b}_1+{X}_1+{b}_2{X}_2+\cdots {b}_n{X}_n+\varepsilon \right)} $$

The dependent variable (social security enrolment) takes on a value of 1 for a firm enrolled with social security and 0 for a non-enrolled firm. *X*_*1*_ through *X*_*n*_ represent characteristics of firms and of company heads; *bi* represents the coefficient for the respective *X* variable; and ε represents the error term, which includes unobserved variables. The logit model is used to estimate the odds ratios, which represent the odds that a firm will enrol in social security given a certain characteristic, when other covariates are held constant. The odds of enrolling in social security are given by the notation below:

$$ OR=\frac{P_{\mathrm{SS}}}{1\mathit{\hbox{-}}{P}_{\mathrm{SS}}} $$

Sampling weights were applied to the logit model to account for the stratified random sampling approach. The post-survey weighting adjusts for the probability that the firm was selected and also accounts for the refusal rates.^3^ This post-survey weighting restores the proportions in the sample to the proportions in the SSO sampling frame [20]. Tests for collinearity among independent variables were performed in Stata and are presented with the results.

As is often the case with firm surveys, the refusal rate was quite high.^4^ Among the firms that were contacted, 20 % declined to participate in the survey, although there was no significant difference in participation between member and non-member groups. In an additional 13 % of firms, the interview could not be successfully arranged (after attempting to follow-up or re-schedule three times). Therefore we replaced a total of 33 % of the sample (43 out of the 130 firms) using the original sampling approach.

Qualitative information from open-ended responses was first translated and transcribed and then coded in Excel. Findings were analysed using thematic analysis [21, 22] and were used to help interpret and validate the quantitative results.

## Results

### Determinants of enrolment in social security

#### Background characteristics of sample

Table 1 outlines background characteristics of the sample by insurance status. Significantly more SSO member firms were state-owned, and under foreign ownership or mixed ownership, while the majority of uninsured firms were domestically owned. SSO member firms also had a significantly larger workforce relative to their non-member counterparts but there was no significant difference in gender balance or the proportion of permanent employees. Although similar with respect to assets, a higher proportion of SSO member firms had higher company revenues, were more likely to be members of a business organization, to have company heads who are foreign-born, and to have attended post-secondary education.

**Table 1:** Background characteristics of sample

Firm characteristics	SSO (*n* = 65)	Non-SSO (*n* = 65)	*p*-value
*Industry category (% of firms)*			
Manufacturing	45.9	29.0	0.0084**
Construction	2.4	24.0	
Trade	17.7	10.6	
Services	34.1	36.5	
*Total*	*100*	*100*	
*Ownership (private/state)*			
Ownership 1: State-owned or partially state-owned (ref = 100 % private)	24.7	2.5	0.005**
Ownership 2 (% of firms):			
100 % domestic	53.3	88.2	0.001**
100 % foreign	24.3	9.4	
Mixed partnership (domestic & foreign)	22.4	2.4	
*Total*	*100*	*100*	
*Employees*			
Mean # of permanent employees	144.11	53.01	0.068*
Mean # of temp employees	55.53	8.98	0.071*
Mean # of daily wage employees	5.41	1.21	0.121
Permanent employees as a % of workforce	78.3	76.7	0.230
% of workforce female	37.7	38.8	0.856
*Size of company (financial)*			
Company Assets (2008) (%)			
< 1 billion kip	31.3	35.0	0.184
1-10 billion kip	43.1	53.3	
> 10 billion kip	25.6	11.7	
Company Revenues (2008) (%)			
< 1 billion kip	31.9	54.7	0.036**
1-10 billion kip	40.5	36.1	
> 10 billion kip	28.0	9.2	
*Other firm characteristics (%)*			
>1 service outlet/factor/branch/store	71.8	79.1	0.429
Member of business organization	55.8	29.8	0.011**
Higher than average risk	12.7	16.1	0.628
Age distribution (at least 70 % of employees are <35 years)	67.4	69.1	0.868
Mean company turnover (annual)	8.5	8.5	0.982
Level of tax payment			
central	48.2	20.1	0.011**
provincial	40.9	46.2	
district	11.0	34.0	
*Characteristics of company head (%)*			
Nationality (head is Laotian)	64.0	86.8	0.008**
Mean age (years)	51.8	49.3	0.307
Gender (head is male)	85.3	76.7	0.296
Education of head (attended university/college or higher)	86.3	57.6	0.003**

**significant at 5 %; *significant at 10 %. Reported results are based on t-tests of means for continuous variables and chi-squares for proportions/categorical variables. All estimates are weighted to account for design effect and non-response

#### Which employer characteristics are associated with enrolment in social security?

The relationship between firm characteristics and enrolment in social security, when all other factors are held constant, are presented as odds ratios in Table 2. The odds of enrolling were significantly higher for firms in the trade industry relative to the services industry. Ownership was also associated with enrolment: the odds of enrolling were approximately 16 times higher for state-owned enterprises than for private firms, which is not surprising given that social security is a government-mandated program.^5^ Although foreign owned companies were not significantly more likely to enroll, the odds of a mixed company (where ownership is shared between domestic and foreign owners) enrolling was 24 times greater than the odds of a domestic company enrolling. It is possible that mixed companies, due to the nature of their business, receive pressure to comply with industry regulations, or that these types of firms have a stronger compliance culture.

**Table 2:** Odds ratios of enrolment, by firm characteristic

	Odds ratio	St. error	z-statistic	*p* > |z|
Industry type (Reference group: services)				
Manufacturing	1.149	0.769	0.21	0.836
Construction	0.256	0.275	−1.27	0.205
Trade	4.469	3.371	1.99	0.047**
Ownership 1: State-owned or partially state-owned (ref: 100 % private)	16.891	19.578	2.44	0.015**
Ownership 2: (Reference group: 100 % domestic)				
100 % foreign (vs. domestic)	6.782	10.141	1.28	0.201
mixed (vs. domestic)	24.020	23.557	3.24	0.001***
Size of permanent workforce =20-59 employees (ref: 0–19 emp)	1.492	0.960	0.62	0.535
Size of permanent workforce = 60+ employees (ref: 0–19 emp)	3.296	2.308	1.7	0.088*
Revenues (Reference group: <1 billion kip)				
1-10 billion Kip	1.437	0.788	0.66	0.508
> 10 billion Kip	1.210	0.913	0.25	0.800
Firm has higher than avg perceived risk (3+ on a scale of 1–5)	0.621	0.635	−0.47	0.641
More than one service outlet/branch/factor/store	1.357	0.856	0.48	0.629
Member of business organization (ref: no membership)	1.945	1.048	1.23	0.217
Employs any temporary workers	1.195	0.653	0.33	0.744
At least 10 % employee turnover (annually)	0.476	0.280	−1.26	0.207
Taxes paid at central level (ref: taxes paid at lower levels)	0.743	0.522	−0.42	0.672
Head of company is Laotian (vs. foreign)	2.178	2.590	0.65	0.513
Head of company has a university education or higher	3.426	1.821	2.32	0.021**
Head of company is male	0.626	0.404	−0.73	0.468

*significant at 10 %; **significant at 5 %; ***significant at 1 %

Prior to the interviews, key informants reported that they expected enrolment to be higher for larger firms. The study confirmed that larger firms (at least 60 employees), were three times more likely to enroll than smaller firms (less than 20 employees). However, a firm’s decision to enroll was not significantly influenced by the firm’s financial status, perceived risk, number of service outlets, the hiring of temporary workers or employee turnover. Although key informants expected pressure from leaders of business organizations to influence members to enrol in SSO, this claim was not supported by the study findings. The findings also show that the odds of enrolling was more than three times higher in companies whose heads have a university education or higher but nationality and gender had no influence on enrolment.^6^

### Descriptive findings about firms’ behaviour and motivation

#### What motivates employers to enrol/not enrol in social security?

To better understand employers’ decisions to enrol, employers were asked to rate a number of possible reasons for enrolment/ non-enrolment on a scale of one to five, with one being not at all important and five being very important.^7^ The results, summarized in Table 3, confirm the trends identified through multivariate analysis but also shed further light on employers’ decision-making process. Among the insured, the most important reason for enrolment was to ensure employees are covered with health insurance. Increasing employee satisfaction, and improving the health and well-being of employees were also important factors. These findings indicate that employers were most concerned with the health insurance benefits within the social security scheme. In another survey question, respondents ranked health care as the most important benefit, followed by retirement benefits and sick leave. The finding that pressure from the SSO was not an important factor was not surprising given that SSO is not currently enforcing enrolment.

**Table 3:** Most important reasons for enrolment/ non-enrolment

Reasons for enrolment	Rating (1–5)^a^
To ensure employees have health care coverage	4.28
To increase employee satisfaction	4.11
To improve health and well-being of employees	4.08
To ensure employees have retirement benefits	4.00
Strong pressure from international bodies	3.05
Strong pressure from Social Security Organization (SSO)	2.74
Strong pressure from employees	2.72
Strong pressure from membership organization	2.71
Reasons for non-enrolment	Rating (1–5)^a^
Company benefits are better than social security (SS) benefits	4.11
Do not know much about social security	3.42
Quality of government hospitals not good	3.22
Do not use health care benefits/ staff do not get sick	3.22
Employees do not want SS	3.12
Cost of SSO is too high	2.97
Do not trust that money is used well	2.83
High turnover among employees	2.57
Many temporary employees	2.38
Employees prefer to purchase private insurance	2.35

^a^Ratings were applied using a likert scale, with 1 being least important and 5 being most important

Among the non-enrolled cohort of the sample, the most important reason for non-enrolment was that the company’s benefit package is superior to social security. Other important reasons for not enrolling were: employers’ lack of knowledge of social security; the poor quality of government hospitals; and the fact that employees do not use benefits or do not get sick.

To better understand the process by which a firm decides to enrol in social security, employers were asked to identify the people in charge of making the decision to enrol. Most firms mentioned that the director of the company, the general manager or the owner, and less often the executive board, make the decision to enrol. In a few companies, the personnel manager and all staff members were involved in the decision process.

#### Is the benefit package offered by non-member firms comparable to social security benefits?

**Fig. 1:**
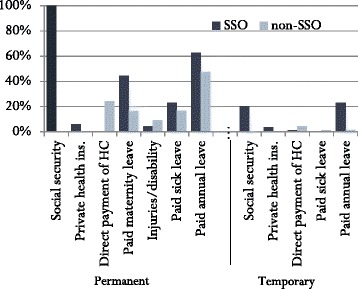
Benefits offered to SSO and non-SSO employees

As stated earlier, the most important reason for not enrolling in social security was that employers offer a better package of benefits than that offered by the SSO. However, the study findings show that the non-enrolled firms offer far fewer benefits to their employees than do SSO member firms (See Fig. 1). In fact, the majority of non-enrolled employers did not offer health care benefits: only one quarter of non-enrolled firms made direct payments for employees’ health care, while none had private insurance. In contrast, some SSO member firms offered extra insurance for programs that were already included in the social security package (e.g., private health insurance, maternity benefits, coverage for injuries, and sick leave). Thus, SSO member employers offered more generous benefit packages to their employees.

With respect to temporary employees, very few in either group received employee benefits. However, among SSO member firms, 20 % offered social security and 23 % paid annual leave to their temporary employees. According to employers, the length of time an employee can work as a temporary employee varies across firms, and 30 % of firms report that there is no limit. Because the majority of temporary employees do not receive benefits, there may be a financial incentive for employers to hire temporary employees to fulfil the duties of permanent employees. The potential for such evasion is discussed in the next section.

#### Are member firms employing strategies to evade social security contributions?

**Fig. 2:**
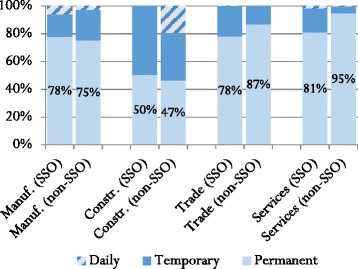
Composition of workforce by industry and SSO status

As stated in the literature review, one of the challenges of implementing and enforcing mandatory insurance is evasion of contributions. In Lao PDR, it was expected that any evasion tactics, such as underreporting the number of employees, reclassifying job descriptions, etc. would be minimal, given that penalties are not yet enacted for non-compliance. Rather than employing deliberate techniques to conceal the fact that they are not enrolling, firms can simply not enrol without consequences. However, one way to detect evasion is to examine the structure of the workforce. A high proportion of temporary workers in non-enrolled firms relative to SSO member firms could indicate that a firm is trying to evade social security contributions by hiring temporary workers, who do not usually receive benefits. Overall, SSO member firms were no more likely than non-enrolled firms to employ temporary workers: in both groups permanent employees represent slightly more than three quarters of the workforce. After controlling for type of industry (See Fig. 2), the difference in the proportion of permanent workers between SSO enrolled and non-enrolled firms is small and not significant in three employment categories. However, in the services industry, SSO member firms employed significantly fewer permanent workers, relative to uninsured firms. Even when differences in the size of workforce, revenues, and ownership (private vs. state-owned; foreign vs. domestic) are taken into account, significant differences still remain. It is possible that firms in the service industry are replacing permanent workers with temporary workers as a means of evading social security payments.

We cannot ascertain from the data in Fig. 2 whether all firms (not just SSO members) are evading payment of benefits. The Labour Law requires all employers to provide sick leave, maternity benefits, health care, occupational disease benefits, and pensions for their permanent workforce, irrespective of their social security status. These expenses must be borne by the employer for firms that do not have social security. Therefore, it is possible that firms (both SSO members and non-members) hire temporary workers, or under-report number of permanent employees as a way of shirking the responsibility and costs associated with purchasing employee benefits. Key informant discussions with the SSO in the initial stages of this study revealed that some companies’ reports on the size of the permanent workforce gradually decline over time when in fact, upon closer inspection by the SSO, the number of employees remains constant. By underreporting the number of employees, firms may feel that both employers and employees benefit: firms decrease their indirect labour costs and employees may be given the option of taking the foregone benefits as cash [23].

**Fig. 3:**
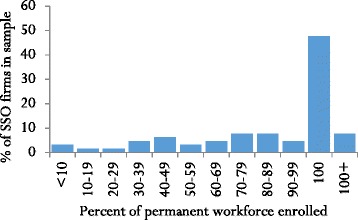
Percent of permanent workforce enrolled with SSO

Prior to the study, key informant interviews suggested that some employers allow employees to opt-out of social security. Given that social security is mandatory for all permanent employees, opt-outs can be considered an evasion strategy used to reduce labour costs. In the international literature, some of the reasons given for evasion at the household or individual level include: a desire to meet current consumption needs; myopic behaviour; and lack of confidence in the scheme (McGillivray, 2001). To further investigate the extent to which opt-outs take place in the Lao social security scheme, we compared data on the number of permanent workers in the company (identified in a module on “employment contracts” in the survey) with the number of permanent employees enrolled in social security (reported in a second module on “employee benefits” later in the survey). If firms are complying with social security, 100 % of the permanent workforce should be enrolled. However, the findings presented in Fig. 3 show that 44 % of member firms enrolled less than 100 % of the permanent workforce in the scheme. In fact, 94 % of employers with social security benefits openly admitted to giving employees the option to enrol.

## Discussion

The background research and the findings from this study contribute new evidence about the enrolment operations of a mandatory social security scheme in Laos and reveal several opportunities and barriers to expanding SHI. Larger firms in the trade industry were the most likely to enrol in social security. Additionally, mixed ownership companies (where ownership is shared between domestic and foreign owners) had a significantly and substantially higher odds of enrolling than a domestic company. Further investigation into whether these mixed ownership companies receive pressure to comply with industry regulations, or have a stronger compliance culture, would help to explain these findings. There was no association between firm enrolment and the level of workplace risk and these findings are consistent with a study in Shanghai, which explored the relationship between firm characteristics and compliance with social security [4].

The finding that companies with higher revenues were more likely to enrol in social security may indicate that social security is more affordable to these firms. Alternatively, staff of larger firms could be more concerned that they will be detected for non-compliance, which could have implications for the company’s reputation even if penalties are not enforced. Although SSO member firms were more likely to have company heads who were more educated or foreign-born, this relationship is unlikely to be a causal relationship but rather to be due to an association with other omitted variables that make it more likely for a firm to comply with the social security law.

The perspectives of employers help to pinpoint some of the motivations for enrolling, or not enrolling, in social security. Firms that enrol in social security are concerned about their employees: employers want to ensure their employees have health insurance; care about employee satisfaction; and want to improve the health and well-being of employees. Although health insurance is the largest and most important benefit in the social security scheme and is the biggest draw to the social security scheme, retirement benefits and sick leave are the second and third most important benefits, respectively. Therefore, it is important to keep in mind that, by design, enrolment in SHI is linked to a broader package of social security benefits and therefore decisions to enrol in social security are determined by the perception of the benefit package as a whole. A study that looked at opportunities to expand social health insurance for formal workers in Vietnam (which is also part of a larger social security scheme) found that reforming pensions, and possibly other benefits, would make social security more attractive to private sector employers and could facilitate the scheme’s expansion [24]. Thus, efforts to move towards UHC may need to go beyond the health sector in countries where social health insurance is integrated with other non-health benefits.

The main reason given for not enrolling was that firms offered a better package of benefits to their employees, although this explanation is discordant with other evidence showing that few non-member firms actually offer benefits to their employees. Other important reasons for not enrolling were that employers are not adequately informed about social security. Although promotion activities targeted at employers could help to encourage enrolment, there are other barriers besides lack of knowledge of the scheme that prevent firms from enrolling. For example, the third most important reason for not enrolling is that the quality of government hospitals is not good. Quality was mentioned elsewhere in the survey as one of the major weaknesses of the social security scheme and appears to be a common theme affecting enrolment in health insurance in Laos. For example, another study on enrolment in CBHI in Laos suggests that quality of hospitals is an important factor affecting enrolment [14]). Thus, it is likely that in the absence of substantial improvements to the health care system, poor quality of care will be a limiting factor in expanding enrolment in all types of insurance – including social security.

Given the problems of evasion documented in social security schemes [1–3] this study explored whether or not firms were evading social security benefits. The findings indicate that there may be some evasion taking place in the services industry, and it is possible that this evasion behaviour is being practiced across all firms, regardless of social security status. As the SSO strengthens enforcement of enrolment, evasion of payments will likely become a bigger concern that will require greater attention. Evasion can lead to a significant loss of revenues for the government. For instance, in Colombia, evasion of social security payments among formal sector workers was estimated to cost US$836 million in forgone revenues in a year (2.75 % of GDP) [25], while in the Philippines and Kazakhstan, only 30 and 40 % of expected revenues from social health insurance, respectively, were actually collected [26, 27].

There are a number of limitations to the study. First, the restriction of the sampling frame to one province and four industries may limit the generalizability of the findings. Second, given that questions about non-compliance with social security are likely to be sensitive, some bias in this study, due to non-response and false-response, is expected, although response rates did not significantly differ between the SSO member and non-member firms. It is difficult to estimate the direction of the bias given that detailed information about the firms that refused to participate is not available. It is possible that the discussion about enrolment in social security, particularly for the non-compliant firms, was a sensitive topic and may have prompted false responses.

Despite the limitations, the results provide evidence regarding the perspectives of private sector employers – a group that is not usually engaged in health sector reform discussions. A number of issues emerge from the study that suggest the need for operational changes that could strengthen the scheme and facilitate expansion. For example, introducing legislation for enforcing enrolment; making the social security benefit package more attractive; and building capacity within the SSO to stimulate and enforce enrolment, could all be effective strategies to ensuring firms enrol in social security. These recommendations are discussed in more detail elsewhere [14, 19]. Additional strategies used internationally, such as issuing warning letters, or benchmarking tax administration across countries to increase accountability could also be considered although approaches will need to be country specific and priorities will differ across countries. It is clear, however, that efforts to strengthen and expand the reach of the social security scheme will require broader changes to make progress towards UHC. For example, there are high costs to stronger enforcement, as offices will need to be established throughout the country and infrastructure and legislation required for enforcement will need to be developed. The costs would likely increase considerably if the SSO begins targeting smaller and less formal firms. Additionally, the forgone revenues associated with a potential increase in informality and evasion must be considered.

Even if social security can play an important social insurance role for the small but growing formal sector in Laos, it is unlikely to be a vehicle for improving access to health services, reducing health-related financial risk on a broad basis, or generating substantial revenues for the health sector. The scheme, which covered 1.5 % of the population at the time of the study, and targets a formal sector that is currently very small, has limited potential for scale-up. The new Social Security law does provide an option for the self-employed to enroll voluntarily but the literature on voluntary insurance shows that such mechanisms are unlikely to achieve high coverage rates. Thus, countries with social insurance schemes that target a small formal sector will need complementary approaches to help them achieve UHC. In Lao PDR, there is scope for building on other health protection schemes, including community-based health insurance and health equity funds. Currently, the different health protection schemes are highly fragmented, although there are plans to merge all schemes into one national social health insurance fund to pave the way for UHC by 2020 [28]. Merging the schemes into one fund would be an important step towards improved coordination and reduced administrative costs. Integration under one organization could also increase purchasing power and facilitate better supervision of quality. However, given the current low coverage rates and the difficulty of enforcing enrolment in the large informal sector, it is unlikely that simply merging the schemes will facilitate expansion of enrolment.

## Conclusions

This study demonstrates the challenge of achieving UHC through social health insurance in countries where the formal sector is large and enforcement capacity is limited. As Laos and other countries address the challenge of low coverage, figuring out how to increase coverage should be accompanied by efforts to improve the supply of health care. Improving quality of health care is particularly important in Laos, where this study and others indicate quality of care as a major reason for poor enrolment. Quality improvements in Laos will therefore require substantial public investment, along with efforts to strengthen public financial management. Successful pathways to UHC – in Laos and other countries - must focus on the demand and supply side, to make sure coverage is worth having and valued by beneficiaries.

## Footnotes

1 Employees and employers contribute 4.5 and 5 % of employees’ salaries, respectively, up to an income ceiling of LAK 1,500,000 ($175.00). Health insurance makes up the largest fund, comprising 4.4 out of 9.5 percentage points of an employee’s earnings.

2 Less than 30 % of targeted firms are enrolled but only a subsample of firms in the country have been targeted given the way the SSO identifies and contacts its target group. The SHI scheme covered approximately 1.5 % of the population at the time of the study.

3 Probability weights were calculated as the inverse of the sampling probability multiplied by the response rate, i.e., (# in the population/# in the sample) X (# sampled/# refused + # sampled). Thus, the weights act as an inflation factor to restore the strata in the sample to their respective proportions in the sampling frame.

4 The low response rate was expected given previous difficulty of interviewing private sector employers in Laos and because the initial point of contact was through telephone.

5 In this sample, all but one state-owned enterprises (SOEs) or partially state-owned enterprises were enrolled in social security. Therefore, the effect of state-ownership on enrollment is very strong. It is not clear whether there is an additional mechanism for SOEs to enroll in social security, but it is possible that SOEs are pressured to enroll or are automatically enrolled.

6 It was suspected that there was some collinearity between nationality and education and between nationality and ownership. However, tests for collinearity were performed in Stata and all variance inflation factors were low (below four). Following guidance in the UCLA Stata help guide, a value lower than 10 is tolerated. Another model was run without the Lao national dummy variable but the significance of other variables did not change. Thus, nationality was left in the model.

7 The possible factors affecting enrolment/non-enrolment were identified by first asking employers to list their most important reasons for enrolling/not enrolling. Respondents were then asked to rank the full list of responses.
